# Not enough by half: NFAT5 haploinsufficiency in two patients with Epstein-Barr virus susceptibility

**DOI:** 10.3389/fimmu.2022.959733

**Published:** 2022-09-27

**Authors:** Daniela Olivia Lopez-Rivera, Lina Maria Castano-Jaramillo, Marco Antonio Yamazaki-Nakashimada, Rosa María Nideshda Ramirez Uribe, Celso Tomás Corcuera Delgado, Karen R. Ignorosa-Arellano, Edgar Alejandro Medina-Torres, Laura Berrón Ruiz, Sara Elva Espinosa-Padilla, Selma C. Scheffler-Mendoza, Gabriel López-Velázquez, Mario Ernesto Cruz-Munoz, Saul O. Lugo Reyes

**Affiliations:** ^1^ Molecular Immunology laboratory at the Faculty of Medicine, Universidad Autonoma del Estado de Morelos, Cuernavaca, Mexico; ^2^ Pediatric Immunology Department, Fundación Hospital de la Misericordia (HOMI) Hospital de la Misericordia, Bogotá, Colombia; ^3^ Clinical Immunology Service, National Institute of Pediatrics, Health Secretariat, Mexico City, Mexico; ^4^ Stem-cell Transplant Unit, National Institute of Pediatrics, Health Secretariat, Mexico City, Mexico; ^5^ Pathology Department, National Institute of Pediatrics, Health Secretariat, Mexico City, Mexico; ^6^ Gastroenterology Department, National Institute of Pediatrics, Health Secretariat, Mexico City, Mexico; ^7^ Laboratory for Biomolecules and Infant health, National Institute of Pediatrics, Health Secretariat, Mexico City, Mexico; ^8^ Immune Deficiencies Laboratory, National Institute of Pediatrics, Health Secretariat, Mexico City, Mexico

**Keywords:** EBV, Epstein-Barr virus, NFAT5 nuclear factor of activated T cells 5, inborn errors of immunity, primary immune deficiency diseases, whole-exome sequence (WES)

## Abstract

**Introduction:**

The transcription factor Nuclear factor of activated T cells 5 (NFAT5), pivotal in immune regulation and function, can be induced by osmotic stress and tonicity-independent signals.

**Objective:**

We aimed to investigate and characterize two unrelated patients with Epstein-Barr virus susceptibility and no known genetic etiology.

**Methods:**

After informed consent, we reviewed the electronic charts, extracted genomic DNA, performed whole-exome sequencing, filtered, and prioritized their variants, and confirmed through Sanger sequencing, family segregation analysis, and some functional assays, including lymphoproliferation, cytotoxicity, and characterization of natural killer cells.

**Results:**

We describe two cases of pediatric Mexican patients with rare heterozygous missense variants in *NFAT5* and EBV susceptibility, a school-age girl with chronic-active infection of the liver and bowel, and a teenage boy who died of hemophagocytic lymphohistiocytosis.

**Discussion:**

NFAT5 is an important regulator of the immune response. NFAT5 haploinsufficiency has been described as an immunodeficiency syndrome affecting both innate and adaptive immunity. EBV susceptibility might be another manifestation in the spectrum of this disease.

## Introduction

Over 7,000 individually rare diseases affect around 8% of the global population (1, 2). Often, rare diseases are congenital (Mendelian and/or monogenic) and manifest early in life with a range of signs and symptoms that may be traced to a single defect in a cell, protein, or pathway (1). Inborn errors of immunity (IEI) are a group of nearly 500 congenital rare diseases with a predisposition to unusual infections with or without hyper-inflammation, autoimmunity, atopy, and malignancies (3).

Epstein-Barr virus (EBV) is an ancient gamma herpesvirus that has co-evolved with mammals for as long as they exist (4). In *Homo sapiens*, EBV is nearly ubiquitous and innocuous, successful at establishing persistent latent infections despite a wide array of immune system components that participate in the defense against herpesviruses (4). A few patients with IEI, experiments of Nature, are susceptible to chronic, severe, or lethal EBV infections (*e.g.*, fatal infectious mononucleosis, chronic-active infection, lymphoma, and hemophagocytic lymphohistiocytosis). In those patients, more than 30 genes have been identified as causing isolated or combined susceptibility to EBV (5, 6), due to genetic lesions affecting pathways of cytotoxicity, apoptosis, MAPK, JAK-STAT, and calcium signaling in lymphocytes (mainly CD8+ T cells, but also Natural killer (NK), NKT, B cells, CD4+), and macrophages.

Nuclear factor of activated T cells 5 (NFAT5), also known as Tonicity enhancer binding protein (TonEBP), is a transcriptional regulator that belongs to the Rel family, which also includes other NFATs (1 through 4) and NFκB. The similarities, however, are mainly structural and limited to the DNA-binding domain. NFAT5 shares an N-terminal Rel-homology domain for DNA-binding and nuclear localization, but lacks the interface (docking sites) for the phosphatase calcineurin that is present in other NFAT proteins, and can thus be activated independently of this calcium/calcineurin signaling cascade (7, 8). It was initially identified as a tonicity-responsive transcription factor, as it is induced upon hyperosmotic stimulus. However, the expression of NFAT5 mRNA in a wide variety of tissues, including but not limited to hypertonic stress, suggested transcription regulation activity besides that induced by osmotic stress (8, 9).

NFAT5/TonEBP can be induced by diverse signaling pathways such as osmotic stress and tonicity-independent (isotonic) receptor-mediated signals, such as *toll*-like receptor (TLR)-activated macrophages and T-cell receptor (TCR)-stimulated T lymphocytes, for distinct transcriptional responses and regulation of immune and cell function (10–12). NFAT5, thus, is also sensitive to ischemia, hypoxia, heat shock, viral/mycobacterial infection, cytokines, and biomechanical stretching, all of which result in activation, upregulation, and nuclear accumulation (13).

In 2015, one patient with early onset sinopulmonary infections and autoimmune enterocolonopathy was found to have a *de novo* heterozygous large genomic deletion in locus 16q22.1 that included NFAT5 and 7 other genes (14). Here, we describe two pediatric patients from Mexico with Epstein-Barr virus (EBV) susceptibility and suspected NFAT5 haploinsufficiency.

## Methods

Cross-sectional descriptive study: case series and literature review. We evaluated two pediatric patients with increased susceptibility to EBV and no known diagnosis.

### Whole-exome sequencing and bioinformatics

Genomic DNA was obtained from whole blood by the salting-out method. Whole-exome sequencing (WES) was performed with an Illumina HiSeq platform (Admera Health, New Jersey), aiming for a 90% coverage of the IDT Xgen library, human genome version 38 (hg38, December 2013), minimum average read depth 50x; processed and analyzed at the National Institute of Pediatrics ID Lab using Galaxy on the cloud (v21.09) (15), Ensembl Variant Effect Predictor, release 104 (16), and Integrative Genome Viewer (IGV) browser (v2.4, Broad Institute) (17).

To retain the variants with the highest genotyping quality, we filtered out those without a minimum MAPQ quality score of 25 and a minimum depth of 10x reads. An average of 10,800 non-intronic non-synonymous variants were called for each patient. The strategy to filter and prioritize germline variants as potentially causative was to include only those variants with a gnomAD exomes minor allele frequency (MAF) of less than 0.001 or “not defined” and, among them, to select those variants with moderate-to-high impact, filtering by variant type (consequence matching “stop”, “frame”, or “splice”), or by pathogenicity prediction (CADD Phred, SIFT, PolyPhen2). Interesting variants were further annotated on Variant Effect Predictor (16) and VarSome (18), as well as visualized using IGV (17).

### Immunological workup and family segregation analysis

In patient 1, we performed flow cytometry for lymphocyte subsets, and carboxyfluorescein succinimidyl ester lymphoproliferation assay as part of her immunological workup. Cell phenotyping was performed in whole-blood samples with anticoagulant (Acid Citrate/Dextrose BD Vacutainer). Lymphocyte populations were stained with the following mixtures of monoclonal antibodies (mAbs): anti-CD45-FITC/anti-CD14-PE, anti-CD3-FITC/anti-CD19-PE/anti-CD45-PerCP, anti-CD4-FITC/anti-CD8-PE/anti-CD3-PerCP, anti-CD3-FITC/anti-CD16+56/anti-CD45-PerCP. To detect *naïve* (CD45RA+) and memory (CD45RO+) T cells, the following antibodies were used: anti-CD45RO-PE/anti-CD45RA-FITC/anti-CD3-PerCP/anti-CD4-APC. To identify B cell subsets, we then stained with a mixture of anti-CD19-APC/anti-IgD-FITC/anti-CD27-PE, anti-CD19-APC/anti-CD38-FITC/anti-CD24-PE, and anti-CD19-APC/anti-CD38-FITC/anti-CD21-PE. Finally, we detected T regulatory cells (Treg) in T cells with anti-CD4-APC/anti-CD127-FITC/anti-CD25-PE. All antibodies were purchased from BD Biosciences, San Diego, CA, USA. Samples were incubated for 30 minutes at room temperature in the dark. After incubation, erythrocytes were lysed by adding FACS lysing solution (BD Biosciences) for 10 minutes. Cells were washed with PBS and fixed in PBS containing 1% formalin.

Sanger sequencing of the exons involved was performed for confirmation and family segregation analysis, as well as cytotoxicity and NK cell maturation assays, at the Molecular Immunology laboratory. Primers for *NFAT5* were: cactgcagATGCTTCTTCAGC (forward) and CTGAGCAGAGCTGCAGTATG (reverse).

### NK cell degranulation assays

NK cell degranulation assays were performed as previously described. Briefly, peripheral blood mononuclear cells (PBMCs, 1x10^6^/ml) were incubated with K562 cells (2x10^6^/ml) in a total volume of 200 μl in a 96-well plate. After 4 hours of incubation at 37°C, cells were recovered and stained using the following antibodies: anti-CD3 FITC, anti-CD56 APC, and anti-CD107 PE (Biolegend, clone H4A3). GolgiStop was not included in these assays.

Cells were acquired on FACSCanto II (BD bioscience) and analyzed with FlowJo 7.6.5 software (Tree Star, Ashland, OR). Gates were set to exclude CD3+ lymphocytes. Thereafter, the percentage of cells positive for CD107a was obtained after gating in CD3-CD56+ lymphocytes. The basal percentages for CD107a were obtained from PBMCs incubated alone. Degranulation was represented as CD107a, which is the difference between the percentage of NK cells expressing surface CD107a after K562 stimulation, and the percentage of NK cells expressing surface CD107a after incubation with medium alone.

### Analysis of NK cells subsets

NK cells were recovered and stained using antibodies: anti-CD3 FITC, anti-CD56 APC, and anti-CD107 PE (Biolegend, clone H4A3). The samples were acquired in a FACSCanto II cytometer (BD bioscience). NK cells were defined as CD3-CD20-CD14-CD56+ cells. The final analysis of the expression for each NK cell marker was performed using FlowJo 7.6.5 software (Tree Star, Ashland, OR) and Infinicyt 2.0.4.

### Structural analysis *in silico*

To interrogate the structural and functional consequences of the variants found, we used AlphaFold (https://alphafold.ebi.ac.uk) to predict the three-dimensional protein structure, and UCSF Chimera (https://www.cgl.ucsf.edu/chimera/) to visualize the structures and domains (19, 20).

## Results

### Case 1

A 7-year-old female with two healthy younger siblings from non-consanguineous parents. At age 5, she began with recurrent episodes of hepatitis, presenting with high-grade fever, abdominal pain, nausea, vomiting, diarrhea, and weight loss, associated with increased transaminases and bilirubin. Serology was negative for hepatitis viruses (HBV, HCV, HAV, and HEV). During the second episode, she was found to have IgG antibodies (IgM-negative) to antiviral capsid antigen (VCA), Epstein-Barr nuclear antigen (EBNA), and early antigen. Quantification of EBV DNA by polymerase chain reaction (PCR) in plasma was persistently positive over several months (328,107 copies/ml), and she was diagnosed with chronic active EBV (CAEBV) infection.

**Figure 1:**
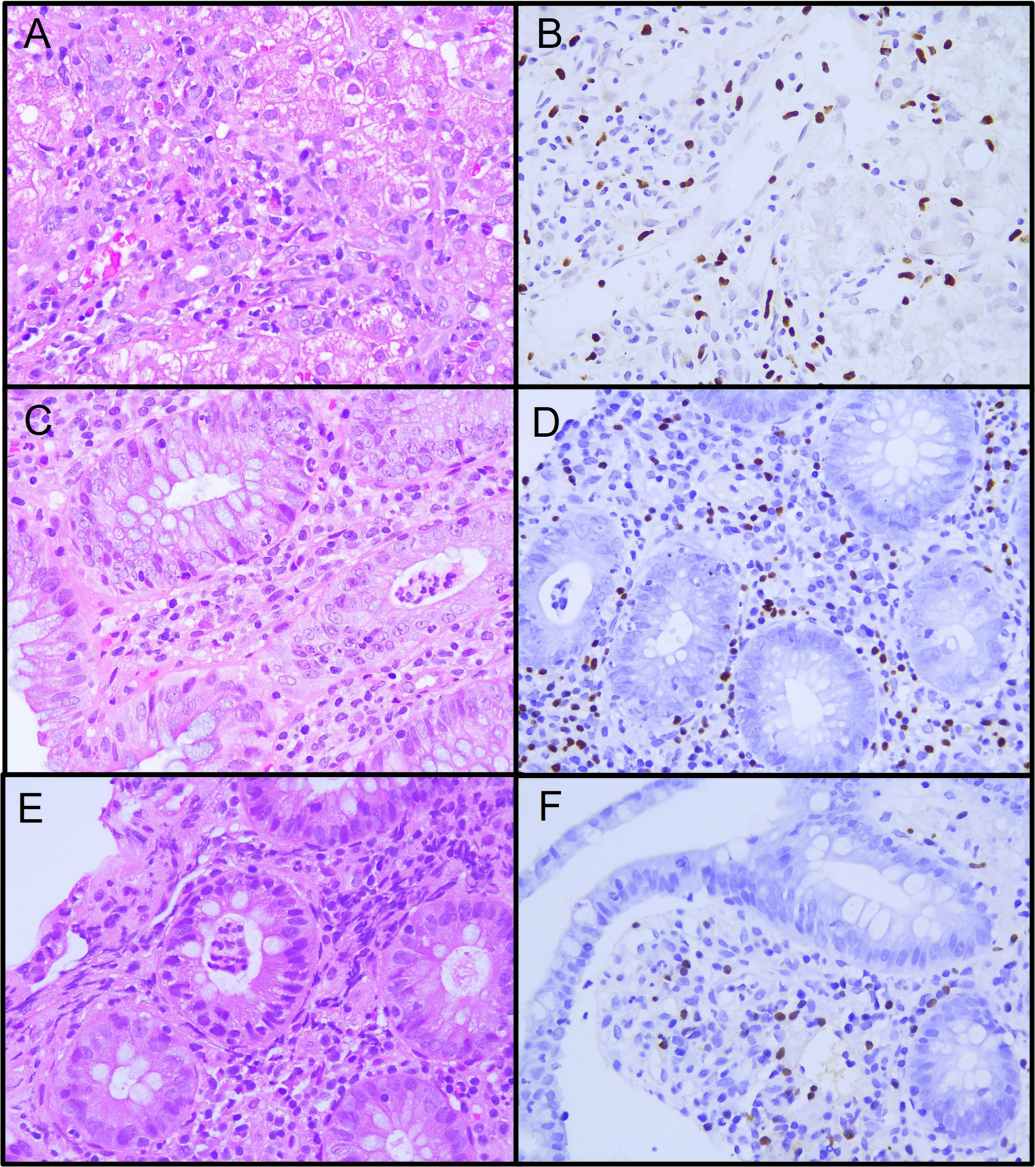
Histopathology specimens from patient 1. **(A)** Liver biopsy showing active diffuse lymphocytic inflammation. **(B)** Epstein-Barr encoding region (EBER) *in situ* hybridization in inflammatory lymphocytic infiltrate. **(C)** Colon biopsy showing intense lymphocytic and polymorphic inflammatory infiltrate at the lamina propria and neutrophilic crypt micro-abscesses. **(D)** EBER positive in inflammatory lymphocytic infiltrate in the lamina propria and crypta. **(E)** Ileum biopsy showing moderate lymphocytic and polymorphic inflammatory infiltrate at the lamina propria and neutrophilic crypt microabscesses. **(F)** EBER positive in inflammatory lymphocytic infiltrate in the lamina propria.

**Figure 2:**
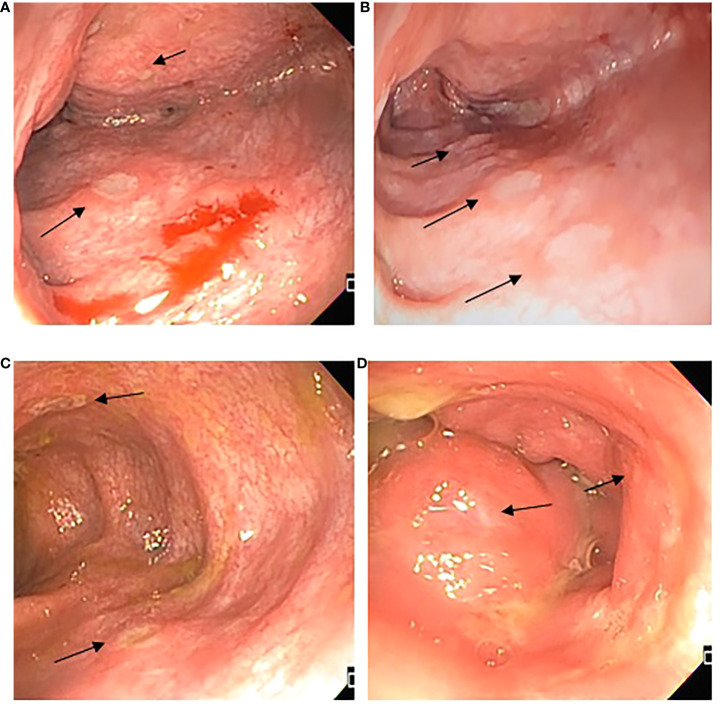
Endoscopic features. **(A)** Ascending colon with edema, erythema, and multiple fibrin-covered ulcers, different sizes between 1 and 5 mm, friability; **(B)** Transverse colon with multiple fibrin-covered ulcers of 3 mm, patchy obliteration of vascular pattern; **(C)** Descending colon presence of deeper ulcers, with mild raise edge; **(D)** Rectum superficial small ulcers <5mm.

The abdominal ultrasound was normal. Cryptosporidium and clostridium were ruled out. A hepatic biopsy showed active diffuse inflammation with lymphocytes, plasmacytes, neutrophils, and eosinophils expanding into the portal space and producing centrilobular necrosis; EBV early RNA (EBER) was positive, predominantly in CD3+CD8+ T cells (**Figure 1**). Colonoscopy revealed pancolitis with ulcers (**Figure 2**), and histopathology showed chronic active enterocolitis associated with EBV, with T-cell predominant EBER; quantification of EBV DNA in the gastrointestinal tract was above 7’000,000 copies per mL. EBV-associated hepatitis and enteropathy were diagnosed.

**Figure 3:**
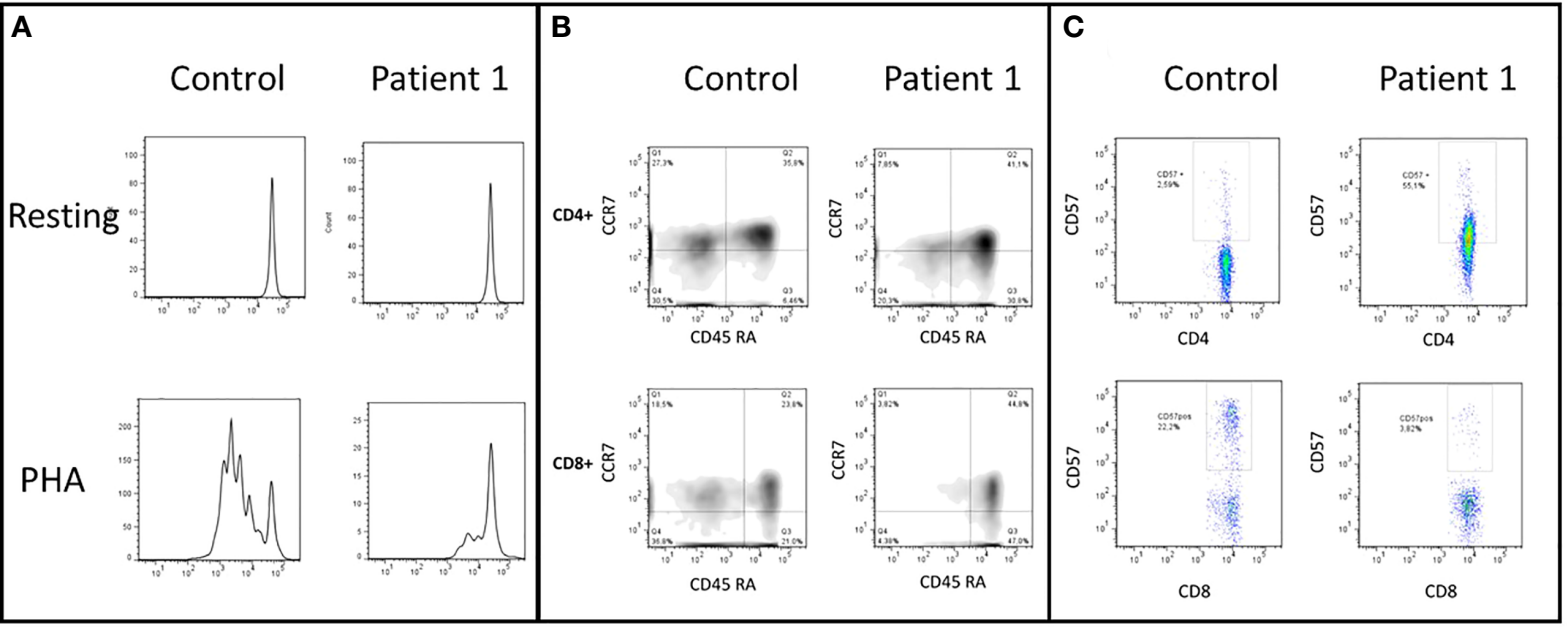
**(A)** CD3+ T-cells lymphoproliferation, resting and after phytohemagglutinin (PHA) stimulus. This test was performed before the HSCT. The patient shows an impaired lymphoproliferation of PHA-stimulated CD3+ T cells. **(B)** CD4+ and CD8+ cells subsets, naive (CD45RA+ and CCR7+), central memory (TCM, CD45RA- and CCR7+), effector memory (TEM, CD45RA-and CCR7-), and CD45RA+ effector memory cells (TEMRA, CD45RA+ and CCR7-). The patient has decreased TCM TEM CD8+ T-cells and expansion of TEMRA CD4+ T-cells. **(C)** CD57+ expression in CD4+ and CD8+ T-cells. Patient has increased senescent CD4+ CD57+ T-cells.

**Figure 4:**
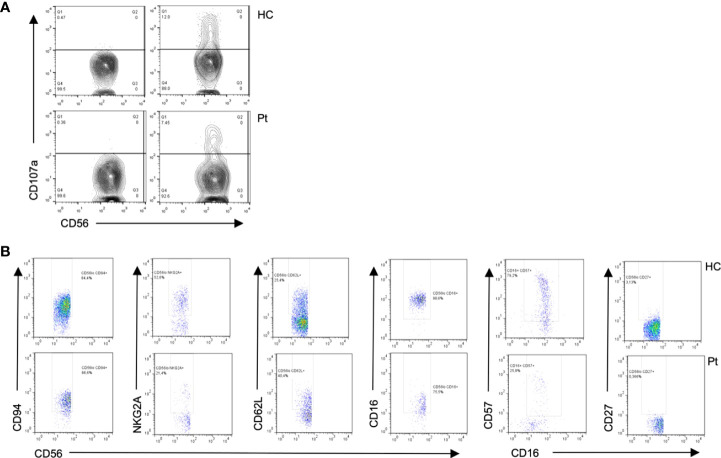
Flow cytometry from patient 1 shows **(A)** Diminished degranulation in Natural Killer (NK) cells from the patient (P) as compared to healthy control (HC). **(B)** Abnormal cell surface marker expressions in NK subsets cells from the patient as compared to healthy control, suggesting impaired NK cell differentiation.

**Figure 5:**
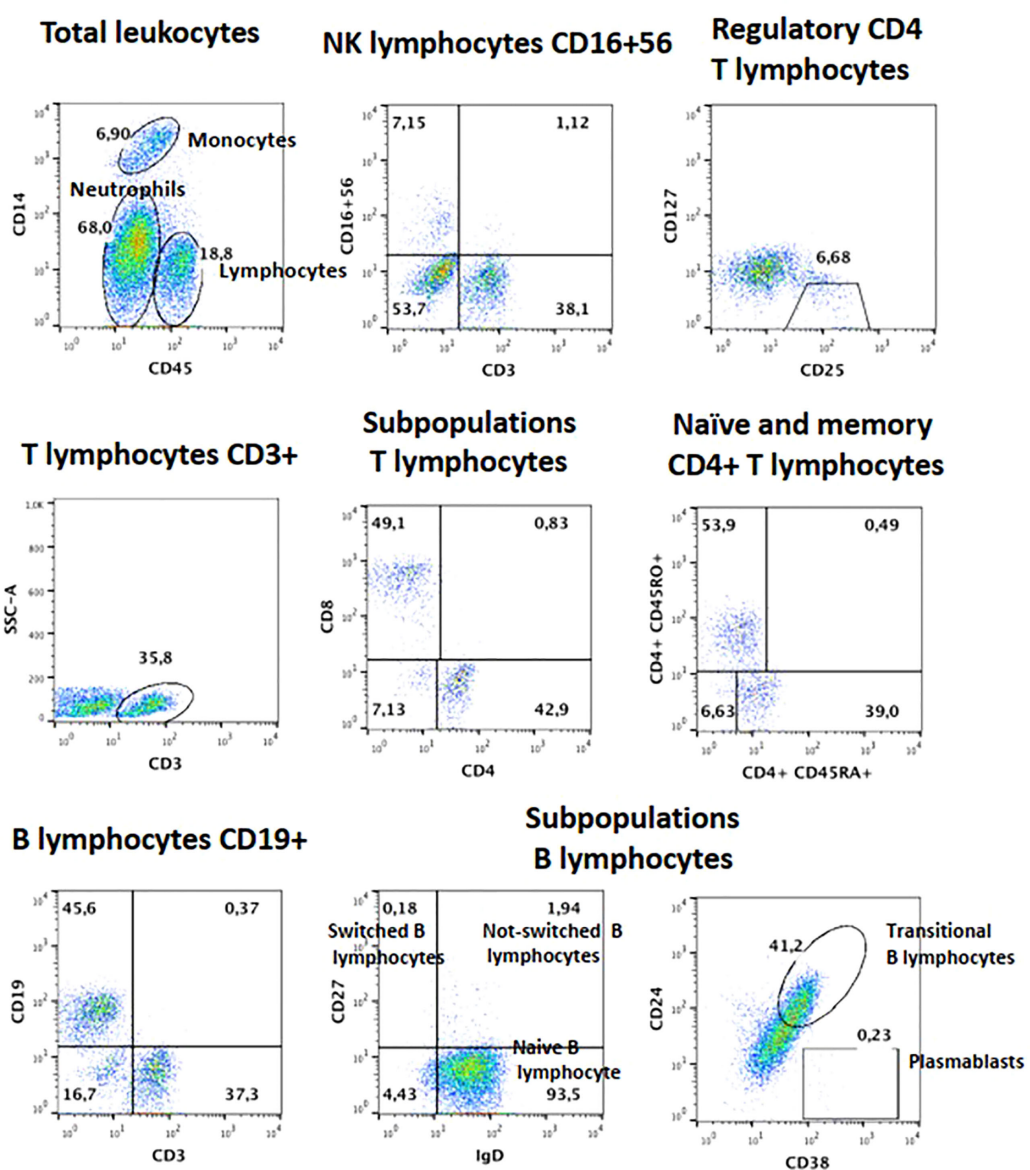
Lymphocyte subsets for B and Treg cells from patient 1, after failed HSCT with mixed chimerism. Plasmablasts and Memory B cells are low, as are Tregs.

Blood counts were normal, with 1,500 peripheral lymphocytes, and 400 monocytes. Serum IgM was marginally low: IgG 1,070mg/dl (normal range 633-1572), IgM 45 (56–352), IgA 126mg/dl (45–236). Lymphocyte subsets by flow cytometry found low T cells: CD3+ 434 cells (1200-2600), CD4+ 294 (650-1500), CD8+ 98 (370-1100); with normal B and NK cells: CD20+ 406 (270-860), CD16/56+ 420 cells (100-480). Further immunological workup revealed an impaired lymphoproliferation of phytohemagglutinin (PHA)-stimulated CD3+ T cells, (**Figure 3A**). Moreover, the patient lymphocyte subsets were shown to have decreased central (CD45RA- CCR7+) and effector (CD45RA- CCR7-) memory CD8+ T-cells, with expanded senescent CD4+ CD57+ T-cells (**Figures 3B, C**). NK cell function was normal based on degranulation assays (**Figure 4A**). In contrast, the expression of various cell surface markers was abnormal in NK cells from the patient as compared to healthy control, suggesting impaired NK cell differentiation (**Figure 4B**). All these assays were performed while the patient was receiving immunomodulatory treatment. Flow cytometry for B cell subsets and Tregs found decreased plasmablasts, memory B cells, and low Tregs (**Figure 5**).

**Figure 6:**
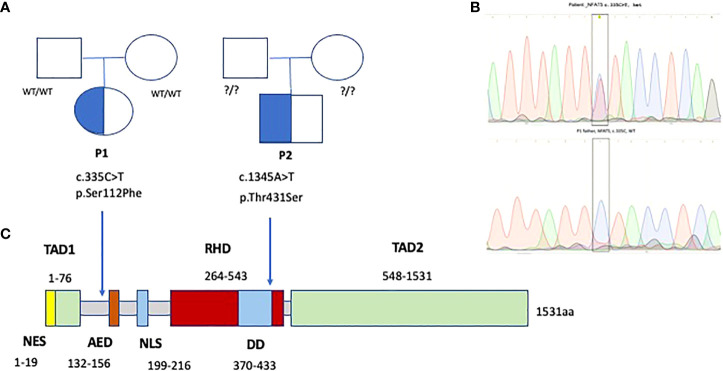
Family trees **(A)** and Sanger electropherograms **(B)** show *de novo* heterozygous missense variants in exons 4 and 7 of *NFAT5*, affecting Transcription activation and DNA binding domains of NFAT5 **(C)**, in two patients with EBV-susceptibility.

Whole exome sequencing (WES) identified a novel (gnomAD allele count 0), heterozygous (MAB 0.51, DP 74x) missense variant in exon 4 (between the transcription activating TAD1 and auxiliary export AED domains) of *NFAT5* (c.335C>T, p.Ser112Phe or p.Ser94Phe), likely pathogenic (SIFT 0, PolyPhen2 0.986, CADD Phred 25.8), at a position highly conserved across species (GERP++ RS 5.67) (**Figure 6**). MutPred Top5 features predict a loss of glycosylation (p=0.007) and loss of phosphorylation at S94 (p=0.019), with a gain of a sheet (p=0.047). Family segregation analysis through Sanger sequencing confirmed the variant to be *de novo*, as both parents had wild-type alleles.

She received treatment with ursodeoxycholic acid, cholestyramine, mesalazine, high-dose intravenous immunoglobulin, enteral immunoglobulin, rituximab, corticosteroids, azathioprine, and cyclosporine, with little improvement; she was later started on tocilizumab with clinical and EBV viral load improvement. The patient underwent allogeneic hematopoietic stem cell transplantation (HSCT) from her haploidentical father, after cyclophosphamide Treg depletion and reduced intensity conditioning with anti-thymocyte globulin, fludarabine, and busulfan. She initially had control of the EBV infection (Undetectable, down from 10,232cp/ml pre-HSCT). She received 4 donor lymphocyte infusions, despite which she evolved to secondary graft failure by day +220. She is currently 8 months post-transplant with mixed micro-chimerism (2.74%) and EBV viral load reactivation (1,531cp/ml), without clinical signs of disease. A timeline of this patient’s evolution and treatment can be found in **Supplementary Materials** as **Figure S1**.

### CASE 2

A previously healthy 16-year-old male presented with hemophagocytic lymphohistiocytosis (HLH) associated with EBV infection. He had two healthy sisters, with no family history of consanguinity. At ages 3 and 6 years, he suffered fissured clavicle, rotula, and forearm fractures associated with traumatism. He also had allergies to penicillin, fava beans, and some fruits. At age 15, he developed a urinary tract infection.

He started at age 16 with fever, cytopenias, elevated triglycerides, and documented hemophagocytosis, for which he was treated with cyclosporine, dexamethasone, and etoposide (21). Soon after discharge, he was readmitted with fever, hepatosplenomegaly, oral candidiasis, and herpetic stomatitis.

Blood counts showed pancytopenia, with Hb 7.5g/dL, white blood cells 2,200 (down to 300), neutrophils 1500 (down to 200), lymphocytes 1,100 (down to 14), monocytes 100, and platelets 19,000-29,000/mm^3^. Ferritin 14,724-225,300 ng/ml. Serum IgM was marginally low, too: IgG 515-1,280 mg/dL (normal range for age 600-1600), IgM 38 (50-190), IgA 272 mg/dL (80-280). IgG1 766, IgG2 154, IgG3 28.3, IgG4 16.9mg/dl. Serum autoantibodies (anti-Ro/La, ANA, lupus anticoagulant), and serologies for HIV, HBV, HCV, syphilis, and brucellosis, were all negative. EBV serum antibodies VCA (IgG), EA, and EBNA were positive. PCR identified 2,580 copies/ml of EBV in serum, and up to 239,411 cp/ml in bone marrow. A bone marrow aspirate (BMA) was normocellular, with megaloblastic changes and active hemophagocytosis. A second BMA found hypocellularity, low megakaryocytes, and 8 hemophagocytes, with EBER diffusely positive in numerous cells.

In addition to the HLH-2004 protocol (etoposide, dexamethasone, cyclosporine) and high-dose intravenous immunoglobulin, the patient received treatment with blood transfusions and filgrastim. He was admitted to the intensive care unit and required mechanical ventilation, despite which he progressed to disseminated intravascular coagulation with multiorgan failure and perished. Given the rapid course of the disease, further immunologic studies could not be performed.

A *post-mortem* WES analysis revealed a very rare heterozygous missense variant, predicted as pathogenic and highly conserved, in exon 7 of 15 of NFAT5, c.1291A>T (p.Thr431Ser), in the DNA-binding domain RHD (Rel homology domain); gnomAD exomes allele count 1 (minor allele frequency MAF 3.99x10^-6^), SIFT 0.04, PolyPhen2 0.996, CADD Phred 24.6 (Minimum significance cutoff 3.3). GERP++ RS 4.85. MutPred Top5 features predict a loss of sheet (p=0.014) and gain of a loop (p=0.024). Unfortunately, after the patient’s death, the family was not willing to undergo family segregation studies. (**Figure 6**).

A table comparing blood counts and acute phase serum reactants of both patients can be found in **Supplementary Materials** as **Table S1**. Tables listing other interesting (rare and likely pathogenic) variants found in the exomes of both patients can be also found as **Tables S2** and **S3**.

### Literature review

A literature review on PubMed Medline searching for: (NFAT5 AND deficiency AND patient) without filters, retrieved 5 results and identified only one previously published human patient with NFAT5 haploinsufficiency (*vide infra*). Our two patients did not share the large heterozygous deletion encompassing 8 genes identified by the authors (14).

### Structural analysis

By using the AlphaFold-predicted structure of NFAT5 that includes the NH-terminal domain (19), we searched for the consequences of missense variant p.Ser112Phe in the human NFAT5 protein (**Supplementary Figure S2**). Thus far the only fragments of the protein to have been successfully crystallized are the middle DNA-binding domains (22). The N-terminal region of the transcription factor is constituted by a large amount of sequence in a random coil configuration, which seems to be rather mobile, intrinsically disordered, and important for nuclear translocation. Importantly, Ser112 is in a disordered region that cannot form enough favorable intra-chain interactions to fold spontaneously but has the energetic capability to gain structure by interacting with co-regulatory proteins. Such coregulatory proteins have been reported as critical for initiating and maintaining transcriptional activity (23). Also, it is important to note that the phosphorylation level of NFAT5 may play a role in its activity. Therefore, the substitution of Ser112 by Phe might be impairing the activity of NFAT5 by diminishing its phosphorylation capacities.

The missense variant p.Thr431Ser was analyzed by using the crystallographic coordinates of the NFAT5 DNA-binding domain (PDB code: 1IMH). Thr431 is in the dimerization domain of NFAT5 (**Supplementary Figure S3**); therefore, the Thr431Ser substitution increases the probability of being phosphorylated and generating a negative charge. Lys131 is near position 431, which in turn enables attraction between charges, increasing rigidity in the dimerization domain (**Supplementary Figure 4**). It has previously been reported that dimerization of NFAT5 is required for proper DNA binding (24).

## Discussion

We present the cases of two pediatric patients with NFAT5 haploinsufficiency and EBV susceptibility: one with CAEBV infection with hepatitis and enterocolitis, and one with fatal HLH. Both patients were found to have rare and conserved heterozygous missense variants in critical domains of NFAT5. The clear limitations of this report are the small number of cases and a lack of mechanistic evidence to prove causation. However, this is the first time that we know, that NFAT5 deficiency has been linked to EBV susceptibility and HLH.

In recent years, NFAT5 has emerged as an important regulator of the immune response. An animal model with homozygous targeted deletion of exons 6 and 7 of the *NFAT5* gene, which encode a critical region of the DNA-binding domain, resulted in complete loss of function and late gestational lethality (25, 26). Heterozygous mice with partial loss of function (haploinsufficiency) resulted in impaired lymphocyte proliferation under hypertonic conditions, lymphoid hypocellularity, and impaired antigen-specific antibody response (25, 27). Lymphoid tissues have a hyperosmolar microenvironment, and lymphocyte-mediated immunity requires adaptation to physiologic osmotic stress; NFAT5 is thus a critical component for optimal adaptive immunity (9), and defective NFAT5 signaling results in poor thymocyte development and survival, independent of NFAT5’s osmoprotective role (27).

In T lymphocytes, NFAT5 is also critical for development and subsistence. NFAT5 is expressed constitutively and abundantly in the thymus and can be induced in mature lymphocytes upon TCR activation (8). In hyperosmotic environments, NFAT5 helps the proliferation and survival of T cells, promotes polarization towards Th17 cells, and attenuates excessive pro-inflammatory responses in T cells (9). NFAT5 deficiency may also contribute to the development and survival of NK cells (14).

There is one report of an NFAT5-haploinsufficient human patient with recurrent sinopulmonary infections during the first years of life, who developed autoimmune entero-colonopathy around age seven. Basic lymphocyte subsets, levels of serum immunoglobulins, and vaccine response were within normal limits, but further immunologic evaluation showed impairment of both innate and adaptive immunity. The patient had an altered distribution of B cell subsets and a selective IgG subclass deficiency. Lymphocyte proliferation to mitogens was normal but antigen-specific proliferation was impaired, and CD8+T cell function was altered due to a reduced ability to degranulate and produce the pro-inflammatory cytokines IFNγ and TNFα. He also had a reduction of CD56+CD16+ NK cells and reduced survival of peripheral blood mononuclear cells (PBMCs) in hypertonic conditions. The genetic evaluation revealed a *de novo* large deletion at 16q22.1 encompassing 8 genes, NFAT5 among them. The authors, Boland et al, used a dominant-negative NFAT5 construct with reduced NFAT5 expression that showed decreased cell viability in hypertonic conditions and reduced production of TNFα by CD8+T cells, analogous to the immune defects seen in the patient (14).

Some other patients from Belarus, Ukraine, and the USA, with NFAT5 haploinsufficiency caused by heterozygous missense or small deletion variants, manifest early in life with diverse autoimmune diseases, and their T cells show reduced proliferation and survival under hypertonic conditions (Svetlana Sharapova, personal communication). Loss-of-function intolerance probability score (pLI) in ExAC and gnomAD is 1.00, with an observed/expected ratio of 0.12 (0.07-0.2), which suggests NFAT5 does not tolerate haploinsufficiency. A high, positive Z-score of 3.13 also predicts an intolerance of missense variants (https://gnomad.broadinstitute.org/gene/ENSG00000102908).

Our two patients from Mexico seem to be the first to present with EBV susceptibility and HLH. NFAT5 is expressed in all T cells, natural killers, and macrophages, the three protagonists of the hemophagocytic cytokine storm. Inside the nucleus, NFAT5 suppresses the induction activity of IRF3, involved in the interferon beta response to viruses (28). Both NFAT5 and EBV directly interact with IRF3: the EBV kinase BGLF4 phosphorylates residues of IRF3 to prevent DNA binding, while NFAT5 forms dimers that compete with the DNA binding of IRF3 to the IFN-β enhancer consensus region (29). This suggests plausibility for the implication of NFAT5 haploinsufficiency in our patients’ phenotypes. Type I interferon is a double-edged sword: unbridled production and prolonged secretion result in systemic inflammation and stem cell exhaustion. Although we do not fully understand the pathogenic mechanisms, lymphocyte maturation/activation defects, excess interferon response, and/or disruption of the latent phase of EBV, are suitable candidates.

In the investigation of patients with increased susceptibility to EBV and HLH, physicians and researchers should also include NFAT5 haploinsufficiency as a differential diagnosis. Phenotypes that include enteropathy, sinopulmonary infections, and reduced numbers of transitional B cells, plasmablasts, and NK cells, are perhaps the most likely candidates.

Next, we want to further characterize the two heterozygous variants in cell lines through plasmid transfection, as one of our patients was successfully transplanted and the other one died. We expect to be able to perform lymphocyte proliferation assay under hyperosmotic conditions, and phenotype rescue.

In conclusion, NFAT5 deficiency can impair T lymphocyte function, and NFAT5 haploinsufficiency has already been described as an immunodeficiency syndrome affecting innate and adaptive immunity. It could also predispose to EBV susceptibility and HLH, given the pivotal role it plays as a transcription factor in lymphocytes, macrophages, and NK cells.

## Data availability statement

The original contributions presented in the study are included in the article/**Supplementary Material**. Further inquiries can be directed to the corresponding author.

## Author contributions

LC-J, MY-N, and SS-M provided health care and clinical information for both patients. DL-R and MC-M performed additional functional immunologic and genetic testing for patient 1 and her family. RRU coordinated the stem-cell transplant and follow-up for patient 1. CCD stained, analyzed, and facilitated pictures of histopathology specimens. EM-T and LBR performed additional experiments. KI-A performed the colonoscopy, described its findings, and retrieved pictures of the procedure. GL-V investigated the potential consequences of both variants in the protein structure. SE-P coordinated the diagnostic approach. SLR performed the processing and analysis of the genetic data. LC-J and SLR wrote the initial manuscript. SS-M and MC-M also designed figures. All authors provided revisions to the initial manuscript, and read, and approved its final version.

## Acknowledgments

Dr. Alfonso Gilberto Ramírez Ristori, MD, from the Pathology Department at the National Institute of Pediatrics (INP) stained, analyzed, and retrieved pictures of histopathology specimens from patient 1, under the supervision of CTCD. We thank Drs Svetlana Sharapova and Vivien Béziat for helpful discussions and feedback on *NFAT5* and haploinsufficient patients. Dr. Sharapova also shared with us her data on other NFAT5 patients and the hyperosmotic assay from her lab in Belarus. The Immune deficiencies laboratory is a Jeffrey Modell Diagnostic Center. Fundacion mexicana para niñ@s con inmunodeficiencias (FUMENI) contributed with sample shipping. Funds for sequencing were obtained through CONACYT: innovation stimulus program and Frontier Science grant #10869.

## Conflict of interest

The authors declare that the research was conducted in the absence of any commercial or financial relationships that could be construed as a potential conflict of interest.

## Publisher’s note

All claims expressed in this article are solely those of the authors and do not necessarily represent those of their affiliated organizations, or those of the publisher, the editors and the reviewers. Any product that may be evaluated in this article, or claim that may be made by its manufacturer, is not guaranteed or endorsed by the publisher.

## Supplementary material

Supplementary Figure 1 A timeline showing treatments and copynumbers of EBV in serum of patient 1.

Supplementary Figure 2 3D structure of the NFAT5 dimer bound to a DNA double helix. PDB code: 1IMH. Structure displayed with UCSF Chimera (Pettersen, et al., 2004).

Supplementary Figure 3 Structural localization of the Thr431Ser substitution, affecting the dimerization domain of NFAT5, close to Lys131. The Serine substitution generates a negative charge and that increases the probability of phosphorylation, which in turn might impair the dimerization of NFAT5 and its interaction with DNA..

## Abbreviations

BMA, bone marrow aspirate; CAEBV, chronic active Epstein-Barr virus; CD, cluster of differentiation; DNA, deoxyribonucleic acid; EBV, Epstein-Barr virus; EBER, EBV early RNA; EBNA, Epstein–Barr virus nuclear antigen; HLH, hemophagocytic lymphohistiocytosis; NFAT5, nuclear factor of activated T cells 5; NK, natural killer; PBMC, peripheral blood mononuclear cell(s); PCR, polymerase chain reaction; TCR, T cell receptor; TLR, toll-like receptor(s); TNF, tumor necrosis factor; VCA, viral capsid antigen; WES, whole-exome sequencing.
