# Supplementary figures and images for: Not enough by half: NFAT5 haploinsufficiency in two patients with Epstein-Barr virus susceptibility
Source: Front Immunol. 2022 Sep 27;13:959733. doi: 10.3389/fimmu.2022.959733 (PMC9552184; doi:10.3389/fimmu.2022.959733)

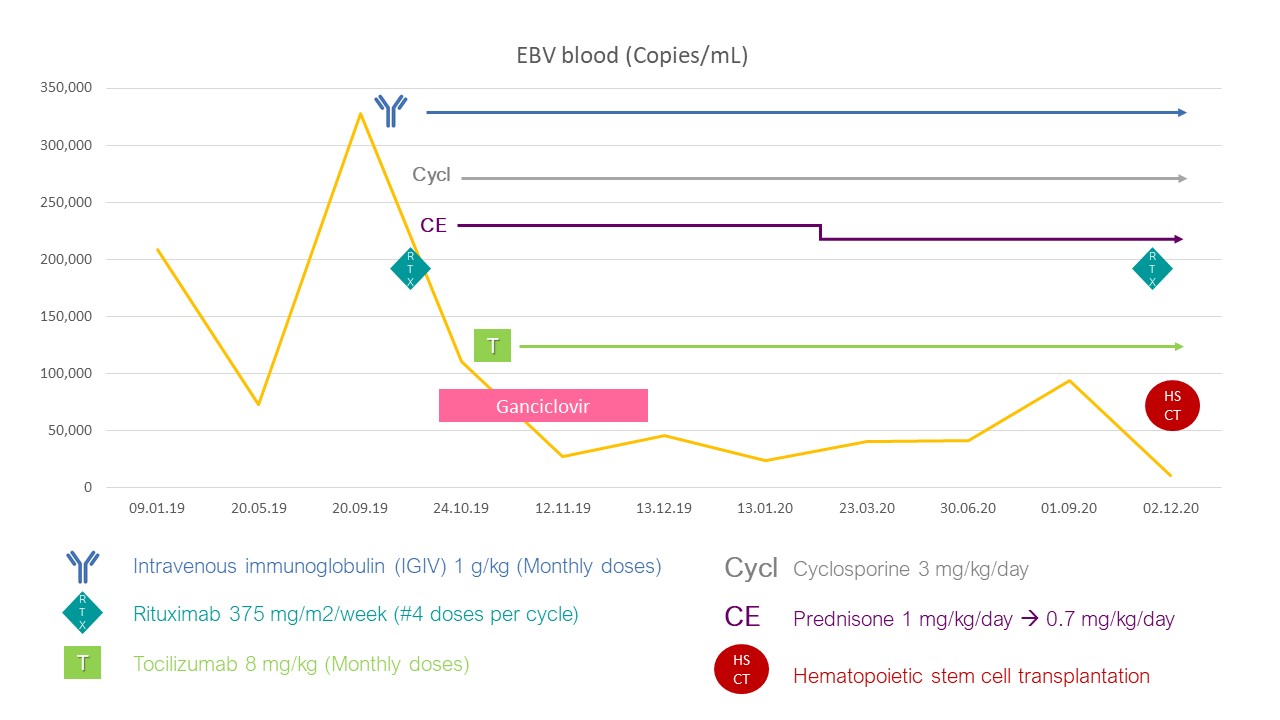

Supplement: Supplementary Figure 1 — A timeline showing treatments and copynumbers of EBV in serum of patient 1. [file Image_1.jpg]

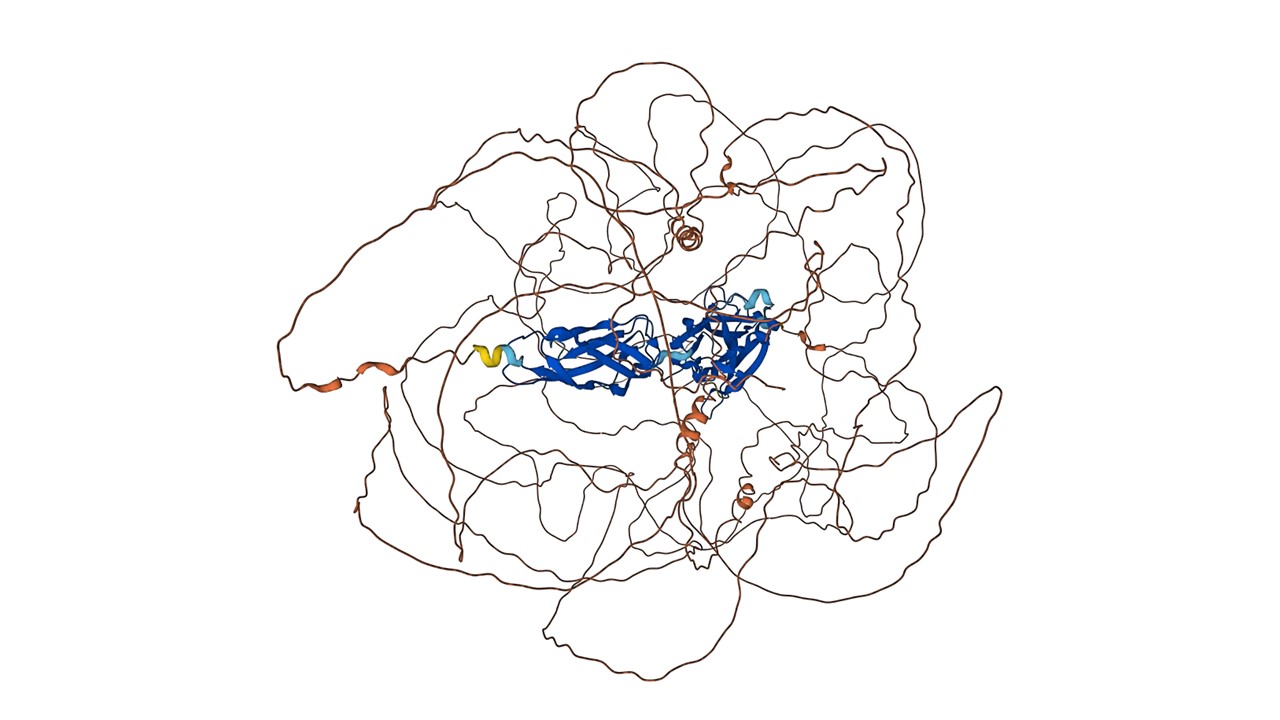

Supplement: Supplementary Figure 2 — 3D structure of the NFAT5 dimer bound to a DNA double helix. PDB code: 1IMH. Structure displayed with UCSF Chimera (Pettersen, et al., 2004). [file Image_2.jpg]

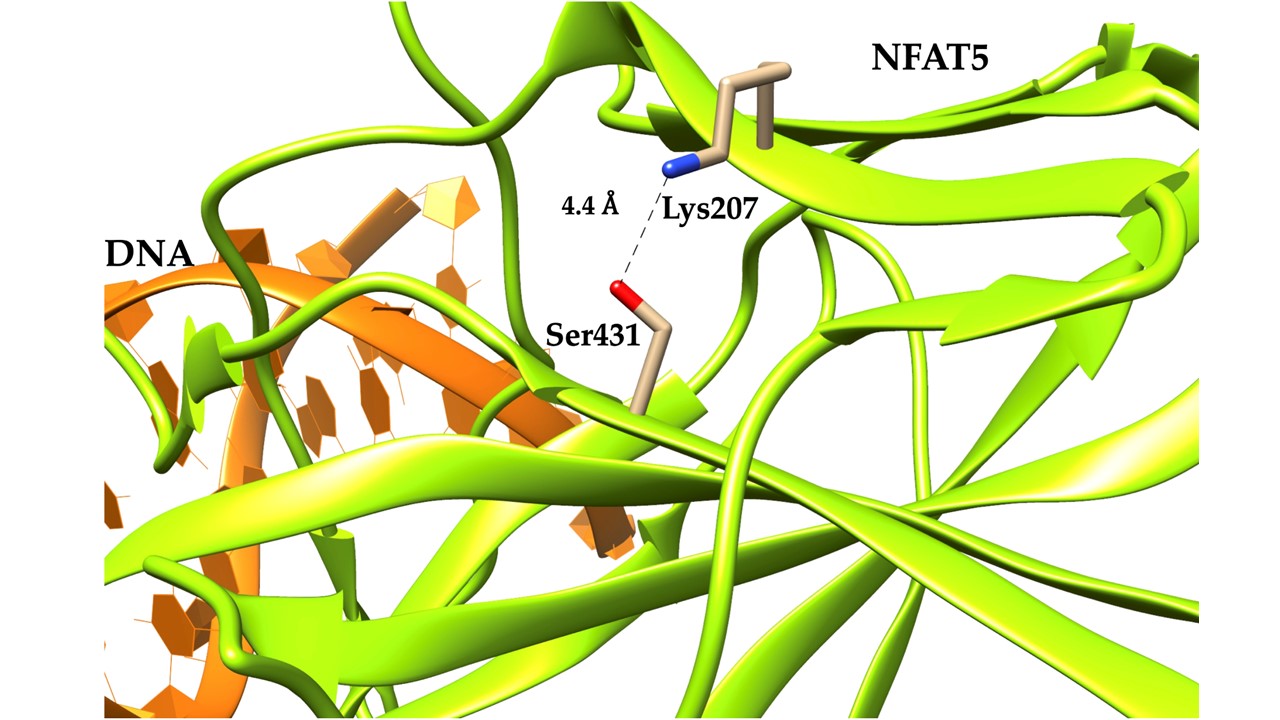

Supplement: Supplementary Figure 3 — Structural localization of the Thr431Ser substitution, affecting the dimerization domain of NFAT5, close to Lys131. The Serine substitution generates a negative charge and that increases the probability of phosphorylation, which in turn might impair the dimerization of NFAT5 and its interaction with DNA.. [file Image_3.jpg]

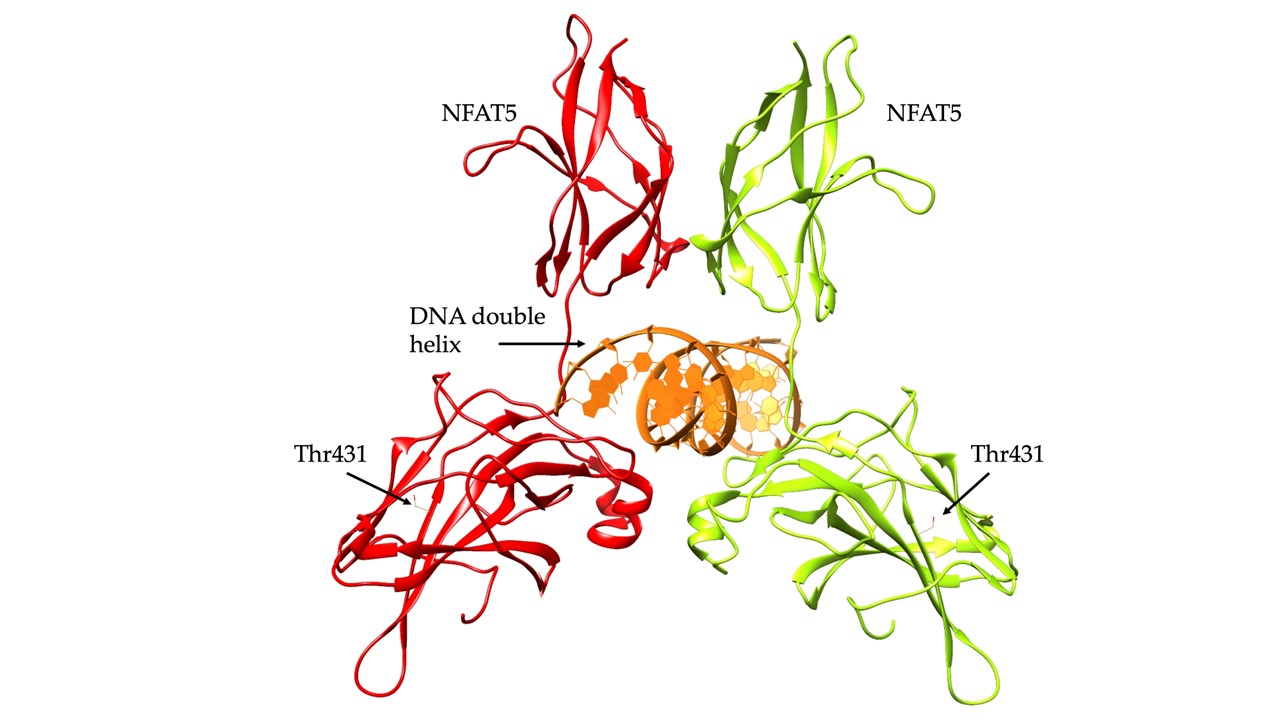

Supplement: Supplementary file 4 [file Image_4.jpg]
