# Supplementary material for: Not enough by half: NFAT5 haploinsufficiency in two patients with Epstein-Barr virus susceptibility
Source: Front Immunol. 2022 Sep 27;13:959733. doi: 10.3389/fimmu.2022.959733 (PMC9552184; doi:10.3389/fimmu.2022.959733)
Supplement: Supplementary file 5 [file DataSheet_1.docx]

| **Gene** | **Zygos MAB** | **Ex** | **cDNA** | **Protein** | **SIFT** | **Poly Phen** | **CADD Phred** | **gnomAD MAF** | **LoF/GERP++ RS** | **Comment** |
| --- | --- | --- | --- | --- | --- | --- | --- | --- | --- | --- |
| **NFAT5** | 0.64 | 4 | 335C>T | Ser112Phe | 0 | 0.986 | 25.8 | 0 | .439/5.67 | **AD IBD and PID** |
| ADORA2B | 0.59 | 2 | 550T>A | Tyr184Asn | 0 | 0.999 | 29.0 | 0 | .607/5.62 | (ADA) |
| **SLC29A3** | Heter | 2 | 146G>C | Arg49Pro | 0 | 0.949 | 23.9 | 0.00019 | .595/3.38 | AR Histiocytosis-lymphadenopathy plus syndrome |
| **SLC29A3** | Heter | 6 | 976A>G | Ile326Val | 1 | 0.02 | 0.515 | 0.82 | .595/-9 | AR Histiocytosis-lymphadenopathy+ |
| DOCK4 | Heter | 31 | 3306C>G | Asn1102Lys | 0 | 0.969 | 25.0 | 6.03e-05 | .717/3.0 | DOCK. Autism |
| CFHR5 | Heter | 10 | 1697C>T | Pro566Leu | 0 | 1 | 22.4 | 5.571e-5 | .613/4.62 | (C3) AHUS |
| ALG2 | Heter | 1 | 299A>G | Tyr100Cys | 0.01 | 0.992 | 24.8 | 4.354e-5 | .389/4.9 | AR CDG2, myasth. |
| MPP5 | Heter | 12 | 1516C>T | Arg506Cys | 0 | 0.982 | 27.8 | 1.24e-05 | .451/5.58 | P55 TCR NFkB |
| IFNL3 | Heter |  |  |  | 0.07 | 0.809 | 14.03 | 0.00020 | --/-0.6 | IFN lambda |
| ITGAD | Heter | 7 | 686C>T | Thr229Met | 0 | 0.998 | 22.8 | 5.171e-5 | .604/3.63 | (ITG2B) |
| DOCK10 | Heter | 36 | 4030A>T | Ile1344Phe | 0 | 0.993 | 27.4 | 0.000449 | .599/5.74 | DOCK. Platelet pr. |
| STK10 | Heter | 14 | 2210G>T | Arg737Leu | 0 | 0.8 | 34 | 0.00012 | .868/5.12 | STK. Testic. tumor |
| ERCC6L2 | Heter | 6 | 1173G>C | Leu391Phe | 0 | 0.997 | 24.9 | 0.00049 | --/3.89 | Bone marrow fail. |
| BTN3A1 | Heter | 10 | 1146T>A | Tyr382* | NA | NA | 24.3 | 0.00047 | .987/-3.8 | T cell act, IFNG |
| DUSP6 | Heter | 2/3 | c.430T>G | p.Ser144Ala | 0.1 | 0 | 22.7 | 0.19 | .07/4.96 | (STK4) |
| DUSP6 | Heter | 1/3 | c.164A>C | p.Asn55Thr | 0 | 0.661 | 27.6 | 4.08e-06 | .07/5.24 | (STK4) |
| BRCA2 | Heter | 15 | 7559G>A | Arg2520Gln | 0 | 0.028 | 24.2 | 3.584e-5 | .09/4.63 | (BLM) breast ca. |
| PRKCSH | Heter | 3/18 | 171C>G | Asp57Glu | 0 | 1 | 25.5 | 0 | .445/2.7 | (PTPRC) AD Poly-cystic liver dis. |
| WWP1 | Heter | 20/25 | 2176G>A | Val726Ile | 0.09 | 0.879 | 23.0 | 9.966e-5 | .389/5.37 | (ITCH) |

**Supplementary material. Table 1 and 2**. Other genes with interesting variants in the whole-exome sequencing analysis of the two patients:

UP: Patient 1 (7 yo female)

DOWN: Patient 2 (7yo male)

| **Gene** | **Exon** | **cDNA** | **Protein** | **Zygos** | **SIFT** | **PolyPh** | **MAF** | **CADD** | **GERP++ RS** | **LoF** | **Comment** |
| --- | --- | --- | --- | --- | --- | --- | --- | --- | --- | --- | --- |
| REN | 3/10 | 356G>A | Arg119His | Heter | 0.04 | 0.622 | 2.88e-5 | 25.6 | 3.47 | .728 | Renin |
| **NFAT5** | 6 | 1291A>T | Thr431Ser | Heter | 0 | 0.996 | 3.99e-6 | 24.6 | 4.85 | .439 | **NK maturation** |
| **NLRC5** | 43/49 | 5077C>G | Leu1693Val | Heter | 0.06 | 0.963 | 0 | 22.2 | 2.76 | -- | **Virus response** |
| VWF | 28/52 | 4123C>T | Pro1375Ser | Heter | 0.04 | 0.996 | 0.00015 | 24.5 | 4.08 | .030 | Von Willebrand |
| IL11 | 5 | 520G>A | Ala174Thr | Heter | 0.13 | 0.812 | 5.18e-5 | 23.3 | 4.63 | -- | craniosynost. |
| CD109 | 10/33 | 1052T>C | Ile351Thr | Heter | 0 | 0.889 | 4.08e-6 | 25.2 | 4.12 | 1 | Alloimmune thr |
| IL31RA | 3/15 | 151T>G | Leu51Val | Heter | 0.02 | 0.453 | 0.00042 | 23.5 | 1.71 | .917 | Amyloidosis |
| **POFUT1** | 5 | 700T>C | Tyr234His | Heter | 0 | 0.997 | 0 | 29 | 5.84 | .447 | Dowling-Degos |
| **TFRC** | 19 | 2186C>T | Thr729Met | Heter | 0.06 | 0.968 | 0.00025 | 24.2 | 6.06 | .531 | (CD8A) AR CID |
| DOCK5 | 51/52 |  | Pro1822His | Heter | 0.06 | 0.99 | 0.00024 | 23.5 | 5.47 | .874 | (DOCK) |
| TADA1 | 7 | 820C>T | Pro274Ser | Heter | 0.03 | 0.953 | 0.0004 | 24.5 | 5.33 | .775 | (STX11) |
| GIPC1 | 9 | 888C>G | Asn296Lys | Heter | 0 | 0.728 | 0.00080 | 23.7 | 2.12 | .098 | (TCN2) |
| CHD3 | 39 | 5974G>A | Ala1992Thr | Heter | 0.45 | 0.001 | 8.48e-5 | 17.0 | 5.05 | .014 | (IKZF1) Macroc |
| **ADAM8** | 11/23 | 1068del | Phe357SerfsTer103 | Heter | 0 | NA |  | NA |  |  | (FERMT3) |
| GRIN2A | 14 | 3031C>T | Arg1011Trp | Heter | 0 | 0.998 | 7.56e-5 | 24.5 | 3.31 | .085 | (SH2D1A) Epileptic encephalopathy |
